# Impact of idiopathic pulmonary fibrosis on recurrence after surgical treatment for stage I–III non-small cell lung cancer

**DOI:** 10.1371/journal.pone.0235126

**Published:** 2020-06-29

**Authors:** Myung Jin Song, Dae Jun Kim, Hyo Chae Paik, Sukki Cho, Kwhanmien Kim, Sanghoon Jheon, Sang Hoon Lee, Jong Sun Park

**Affiliations:** 1 Division of Pulmonary and Critical Care Medicine, Department of Internal Medicine, Seoul National University College of Medicine, Seoul National University Bundang Hospital, Seongnam, South Korea; 2 Department of Thoracic and Cardiovascular Surgery, Yonsei University College of Medicine, Seoul, Republic of Korea; 3 Department of Thoracic and Cardiovascular Surgery, Seoul National University College of Medicine, Seoul National University Bundang Hospital, Seongnam, South Korea; 4 Division of Pulmonology, Department of Internal Medicine, Institute of Chest Diseases, Severance Hospital, Yonsei University College of Medicine, Seoul, Republic of Korea; Baylor College of Medicine, UNITED STATES

## Abstract

**Background:**

Idiopathic pulmonary fibrosis (IPF) is an independent risk factor for lung cancer (LC) development; however, its effect on recurrence after curative surgery remains unclear.

**Objectives:**

This study aimed to determine the impact of IPF on recurrence-free survival following curative surgical resection of stage I–III non-small cell lung cancer (NSCLC) and investigate the effects of patient and surgical factors on the risk of recurrence.

**Methods:**

We reviewed retrospectively collected data of patients with surgically resected stage I–III NSCLC from two tertiary care hospitals in South Korea. By propensity score matching, patients with IPF (LC with IPF) were matched to those without IPF (LC without IPF).

**Results:**

In total, 3416 patients underwent surgical resection, and 96 were diagnosed with underlying IPF. In the LC with IPF group, 89.6% patients were men, and the average age was 69.7 years. Sublobar resection was performed more frequently in the LC with IPF group than in the LC without IPF group, while the rate of mediastinal lymph node dissection and dissected node number were lower in the former group. The 5-year recurrence-free survival rate was significantly lower in the LC with IPF group (49.2%) than in the LC without IPF group (69.1%; P<0.001). Multivariable Cox regression analysis revealed that IPF and postoperative stage III were independent risk factors for recurrence.

**Conclusions:**

IPF may increase the risk of recurrence after curative surgical treatment for NSCLC. Close surveillance for recurrence is mandatory for patients with underlying IPF.

## Introduction

Idiopathic pulmonary fibrosis (IPF) is a devastating lung disease characterized by progressive lung scarring and a histopathological pattern of usual interstitial pneumonia (UIP). The median survival period of IPF ranges from 2.5 to 3.5 years [1, 2]. Patients with IPF exhibit a high prevalence of various comorbidities, including chronic obstructive lung disease, pulmonary hypertension, coronary artery disease, and lung cancer (LC) [3].

The reported prevalence of LC in patients with IPF is 2.7%–45.7% [4–8]. Moreover, the relative risk of LC development is approximately eight times higher in patients with IPF than in the general population [9]. Although adenocarcinoma is the most common histopathological type of LC in the general population, patients with IPF are most commonly affected by squamous cell carcinoma, followed by adenocarcinoma [8, 10, 11].

Patients with stage I and stage II non-small cell lung cancer (NSCLC), as well as those with certain stage III NSCLCs, are considered eligible for curative resection. Complete resection, when possible, remains the mainstay of therapy and is associated with the highest probability of long-term survival. However, if patients with LC who were eligible for surgical resection have underlying IPF, several aspects must be considered. Previous studies have shown that the postoperative morbidity and mortality rates for patients with NSCLC are significantly higher if IPF is present, with the main cause of postoperative mortality being acute exacerbation of IPF (AE-IPF) [12–16]. A few studies have reported the long-term survival of patients with NSCLC and IPF, although overall survival was significantly poorer for these patients than for those without IPF [14, 17–20]. However, the effect of IPF on recurrence after curative surgical treatment for NSCLC remains unclear. Therefore, the present study aimed to determine the impact of IPF on recurrence-free survival and overall survival following curative surgical resection of stage I–III NSCLC, and investigate the effects of patient and surgical factors on the recurrence and mortality.

## Methods

### Patients

**Fig 1:**
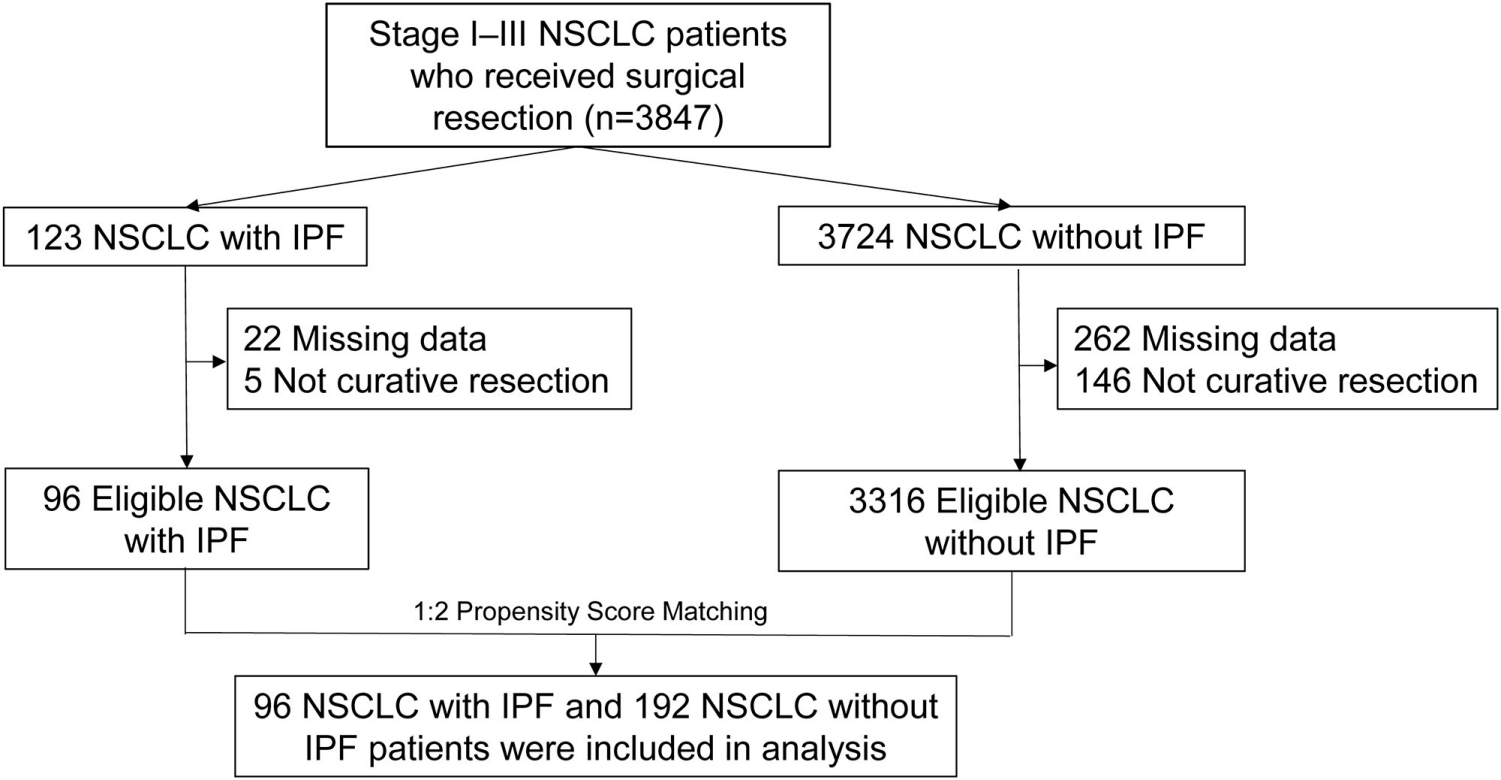
Patient recruitment flow chart.

We retrospectively reviewed the medical records of patients with stage I–III NSCLC who underwent curative resection at Seoul National University Bundang Hospital and Severance Hospital between January 2003 and September 2016. Among these patients, we identified those who were diagnosed with underlying IPF. By propensity score matching of confounding variables, including age, sex, the histopathological subtype of NSCLC, the postoperative stage of NSCLC, and the year of NSCLC diagnosis, patients with IPF (LC with IPF group) were matched to those without IPF (LC without IPF group) in a 1:2 ratio. Flow chart of patient recruitment is shown in Fig 1. This study was conducted in accordance with the amended Declaration of Helsinki. Local institutional review boards and independent ethics committees approved the study protocol (Seoul National University Bundang Hospital, IRB number: B-1707/411-402; Severance Hospital, IRB number: 4-2019-0292).

### Ethics approval and consent to participate

This study was approved by the Institutional Review Board and Ethics Committee of Seoul National University Bundang Hospital and Severance Hospital (Seoul National University Bundang Hospital, IRB number: B-1707/411-402; Severance Hospital, IRB number: 4-2019-0292). Written informed consent was waived as the nature of retrospective study by IRB. The patient’s medical records between January 2003 and September 2016 were accessed. All data were fully anonymized before accessement.

### Definitions

In accordance with diagnostic criteria defined by the International Consensus Statement of the American Thoracic Society and European Respiratory Society in 2018 [1], IPF was diagnosed via a multidisciplinary approach involving pulmonologists as well as radiologists and pathologists specializing in chest diseases.

The LC stage was defined according to the 8th edition of the American Joint Committee on Cancer staging system. Recurrence-free survival was defined as the time from surgery to the first diagnosis of disease recurrence or the last follow-up visit. Death was considered a censoring event for recurrence. Overall survival was defined as the time from initial LC treatment to death or the last follow-up visit. Local recurrence was defined as evidence of tumor cells within the same lobe of the lung or ipsilateral pulmonary hilum, and regional recurrence was defined as evidence of tumor cells within another lobe of the ipsilateral lung or the ipsilateral or subcarinal lymph nodes (LNs), while distant metastasis was defined as evidence of tumor cells in the contralateral lung, contralateral mediastinal LNs, the pleural space, or areas outside the hemithorax.

AE-IPF was defined as acute, clinically significant respiratory deterioration characterized by evidence of new widespread alveolar abnormality, in accordance with the diagnostic criteria proposed by Collard et al. in 2016 [21].

The gender–age–physiology (GAP) index was applied for the assessment of the severity and prognosis of IPF. The index was calculated as follows: gender (0–1 point), age (0–2 points), forced vital capacity (FVC; 0–2 points), and diffusing capacity of the lungs for carbon monoxide (DL_CO_; 0–3 points). Based on the score, IPF was categorized into three stages: I (0–3 points), II (4–5 points), and III (6–8 points). The predicted 1-year mortality rates for GAP stages I, II, and III were 6%, 16%, and 39%, respectively [22].

### Statistical analysis

Propensity scores were estimated through multiple logistic regression analysis with sex, age, the postoperative cancer stage, the histopathological subtype of cancer, and the year of cancer diagnosis as independent variables.

Using the matched set, we examined the effect of IPF on recurrence after curative surgical resection of stage I–III NSCLC. Generalized estimating equations were used to compare continuous and categorical variables of propensity score matched population. Cumulative time-to-event distributions (survival, recurrence) were estimated using the stratified Kaplan–Meier method. Independent predictors for recurrence and overall survival were determined by stratified Cox proportional hazards models. A P-value <0.05 was considered statistically significant. All statistical analyses were performed using SAS software (version 9.4; SAS Inc., Cary, NC, USA).

## Results

### Patient characteristics and surgical methods

In total, 3416 patients with NSCLC underwent surgical resection during the study period, and 96 were diagnosed with underlying IPF. Baseline characteristics of the overall cohort before propensity score matching are shown in Table 1. The LC with IPF group and LC without IPF group showed significant differences in potential confounding factors for recurrence, including age, sex, the histopathological subtype, and the postoperative stage. The average age (69.7 years vs 63.4 years; P<0.001) and proportion of men (89.6% vs 61.8%; P<0.001) were higher in the LC with IPF group than in the LC without IPF group. The most frequent histopathological subtype was squamous cell carcinoma in the LC with IPF group and adenocarcinoma in the LC without IPF group. Patients with IPF showed more advanced postoperative stages (stages II and III) than did those without IPF.

**Table 1:** Baseline characteristics of patients with surgically resected non-small cell lung cancer.

	LC with IPF (n = 96)	LC without IPF (n = 3316)	P-value
Sex (male)	86 (89.6%)	2049 (61.8%)	<0.001
Age (year)	69.7 ± 7.4	63.4 ± 10.0	<0.001
Histology			<0.001
Adenocarcinoma	44 (45.8%)	2262 (68.2%)	
Squamous cell carcinoma	48 (50.0%)	922 (27.8%)	
Adenosquamous	2 (2.1%)	42 (1.3%)	
Large cell	0 (0.0%)	64 (1.9%)	
Sarcomatoid	2 (2.1%)	26 (0.8%)	
Postoperative Stage			<0.001
0	–	74 (2.2%)	
I	43 (44.8%)	2123 (64.0%)	
II	35 (36.5%)	608 (18.3%)	
III	18 (18.8%)	511 (15.4%)	

Values are expressed as the mean ± standard deviation or number (%).

IPF, idiopathic pulmonary fibrosis; LC, lung cancer

Baseline characteristics of the matched groups are shown in Table 2. The diffusing capacity of the lungs for carbon monoxide (DL_CO_) was significantly lower in the LC with IPF group than in the LC without IPF group (83.7±20.8 vs. 104.3±24.7; P<0.001). Other variables, including the location of the primary tumor, forced vital capacity (FVC), and forced expiratory volume in one second, were comparable between groups. In the LC with IPF group, stages I and II as per the GAP index were documented for 94 (97.9%) and two (2.1%) patients, respectively. There was no patient with GAP stage III.

**Table 2:** Baseline characteristics of patients with surgically resected non-small cell lung cancer with or without idiopathic pulmonary fibrosis (propensity score-matched population).

	LC with IPF (n = 96)	LC without IPF (n = 192)	P-value
Sex (male)	86 (89.6)	169 (88.02)	0.602
Age (year)	69.7 ± 7.4	69.0 ± 6.6	0.417
BMI (kg/m2)	24.0 ± 2.8	23.8 ± 3.9	0.503
Smoking exposure			0.642
Never	11 (11.5)	43 (22.4)	
Former	71 (74.0)	107 (55.7)	
Current	14 (14.6)	42 (21.9)	
Smoking (pack-years)	37.1 ± 25.7	34.9 ± 32.1	0.501
Comorbidities			
Hypertension	44 (45.8)	73 (38.0)	0.204
Diabetes mellitus	26 (27.1)	37 (19.3)	0.167
COPD	11 (11.5)	21 (10.9)	0.899
Old TB	18 (18.8)	28 (14.6)	0.346
Other cancer	17 (17.7)	31 (16.2)	0.726
Coronary artery disease	7 (7.3)	24 (12.5)	0.161
Cerebrovascular disease	3 (3.1)	10 (5.2)	0.436
Histology			0.157
Adenocarcinoma	44 (45.8)	92 (47.9)	
Squamous cell carcinoma	48 (50.0)	96 (50.0)	
Adenosquamous	2 (2.1)	1 (0.5)	
Large cell	0 (0.0)	3 (1.6)	
Sarcomatoid	2 (2.1)	0 (0.0)	
Location			0.379
Right upper lobe	16 (16.7)	35 (18.2)	
Right middle lobe	5 (5.2)	11 (5.7)	
Right lower lobe	27 (28.1)	58 (30.2)	
Left upper lobe	23 (24.0)	51 (26.6)	
Left lower lobe	25 (26.0)	37 (19.3)	
Pulmonary function test			
FVC % pred	93.5 ± 13.2	94.7 ± 15.5	0.503
FEV_1_% pred	98.4 ± 15.4	96.6 ± 20.2	0.411
DL_CO_ % pred*	83.7 ± 20.8	104.3 ± 24.7 (n = 153)	<0.001
Postoperative Stage			0.665
I	43 (44.8)	85 (44.3)	
II	35 (36.5)	79 (41.2)	
III	18 (18.8)	28 (14.6)	
Severity of IPF (GAP stage)			
I	94 (97.9)	–	
II	2 (2.1)	–	

Values are expressed as the mean ± standard deviation or number (%).

BMI, body mass index; COPD, chronic obstructive pulmonary disease; DL_CO_, diffusing capacity of the lungs for carbon monoxide; ECOG, Eastern Cooperative Oncology Group; FEV_1_, forced expiratory volume in one second; FVC, forced vital capacity; GAP, gender, age, and physiology; IPF, idiopathic pulmonary fibrosis; LC, lung cancer

*39 patients with missing data in LC without IPF group

The treatment-related characteristics of the matched groups are shown in Table 3. There was no significant difference in the proportion of patients receiving induction therapy and adjuvant therapy between the two groups. Sublobar resection, either wedge resection or segmentectomy, was more frequently performed in the LC with IPF group than in the LC without IPF group, while the rate of mediastinal lymph node dissection was lower in the LC with IPF group (82.3% vs. 99.0%; P<0.001). The mean number of dissected LNs was also smaller in the LC with IPF group than in the LC without IPF group (16.0 vs. 23.2; P<0.001). The two groups showed no significant difference in the resection margin status (R0, complete resection; R1, microscopic residual tumor).

**Table 3:** Treatment-related variables for patients with surgically resected non-small cell lung cancer with or without idiopathic pulmonary fibrosis (propensity score-matched population).

	LC with IPF (n = 96)	LC without IPF (n = 192)	P-value
Induction treatment			0.273
Chemotherapy	1 (1.0)	5 (2.6)	
Concurrent chemoradiotherapy	0 (0.0)	1 (0.5)	
Surgery			
Surgical extent			<0.001
Wedge resection	16 (16.7)	3 (1.6)	
Segmentectomy	8 (8.3)	8 (4.2)	
Lobectomy	70 (72.9)	150 (78.1)	
Bilobectomy	1 (1.0)	18 (9.4)	
Pneumonectomy	1 (1.0)	13 (6.8)	
MLND	79 (82.3)	190 (99.0)	<0.001
Dissected LN	16.0 ± 13.4	23.2 ± 12.8	<0.001
Number of positive LN	1.0 ± 2.3	1.1 ± 2.4	0.629
Resection margin			0.481
R0	92 (95.8)	180 (93.8)	
R1	4 (4.2)	12 (6.3)	
Adjuvant treatment			0.187
Chemotherapy	24 (25.0)	64 (33.3)	
Radiotherapy	3 (3.1)	4 (2.1)	
Concurrent chemoradiotherapy	0 (0.0)	7 (3.7)	
Sequential radiotherapy after chemotherapy	3 (3.1)	5 (2.6)	

Values are expressed as mean ± SD or number (%).

IPF, idiopathic pulmonary fibrosis; MLND, mediastinal lymph node resection; LC, lung cancer; LN, lymph node

### Treatment-related complications

To assess the incidence of treatment-related complications, we defined the duration between the last treatment and the onset of complications as ≤4 weeks (Table 4). The incidence of surgery-related AE-IPF or acute respiratory distress syndrome (ARDS) was significantly higher in the LC with IPF group than in the LC without IPF group. Adjuvant treatment-related complication rates were comparable between groups. Surgery and adjuvant treatment resulted in AE-IPF in 5.2% and 6.7% patients, respectively.

**Table 4:** Treatment-related complications in patients with surgically resected non-small cell lung cancer with or without idiopathic pulmonary fibrosis.

(A) Surgery-related complications			
	LC with IPF (n = 96)	LC without IPF (n = 192)	P-value
Pneumonia	11 (11.5)	25 (13.0)	0.706
Pneumothorax	20 (20.8)	24 (12.0)	0.509
BPF	1 (1.0)	2 (1.0)	1.000
Prolonged MV	2 (2.1)	10 (5.2)	0.224
Arrythmia	1 (1.0)	2 (1.0)	1.000
Bleeding	2 (2.1)	3 (1.6)	0.751
ARDS*	2 (2.1)	2 (1.0)	0.255
AE–IPF	5 (5.2)	–	
ARDS or AE–IPF	7 (7.3)	2 (1.0)	0.012
(B) Adjuvant treatment-related complication (n = 110)			
Postoperative hospital day	10.2 ± 10.0	9.8 ± 11.9	0.780
	LC with IPF (n = 30)	LC without IPF (n = 80)	P-value
Cytopenia	2 (6.7)	17 (21.2)	0.091
GI trouble	8 (26.7)	28 (35.0)	0.409
Pneumonia	6 (20.0)	6 (7.5)	0.071
Pneumonitis	1 (3.4)	1 (1.2)	0.469
AE–IPF	2 (6.7)	0 (0.0)	0.126

Values are expressed as number (%).

*ARDS other than AE-IPF

AE-IPF, acute exacerbation of idiopathic pulmonary fibrosis; ARDS, acute respiratory distress syndrome; BPF, bronchopleural fistula; GI, gastrointestinal; LC, lung cancer; MV, mechanical ventilation

### Recurrence and mortality

**Fig 2:**
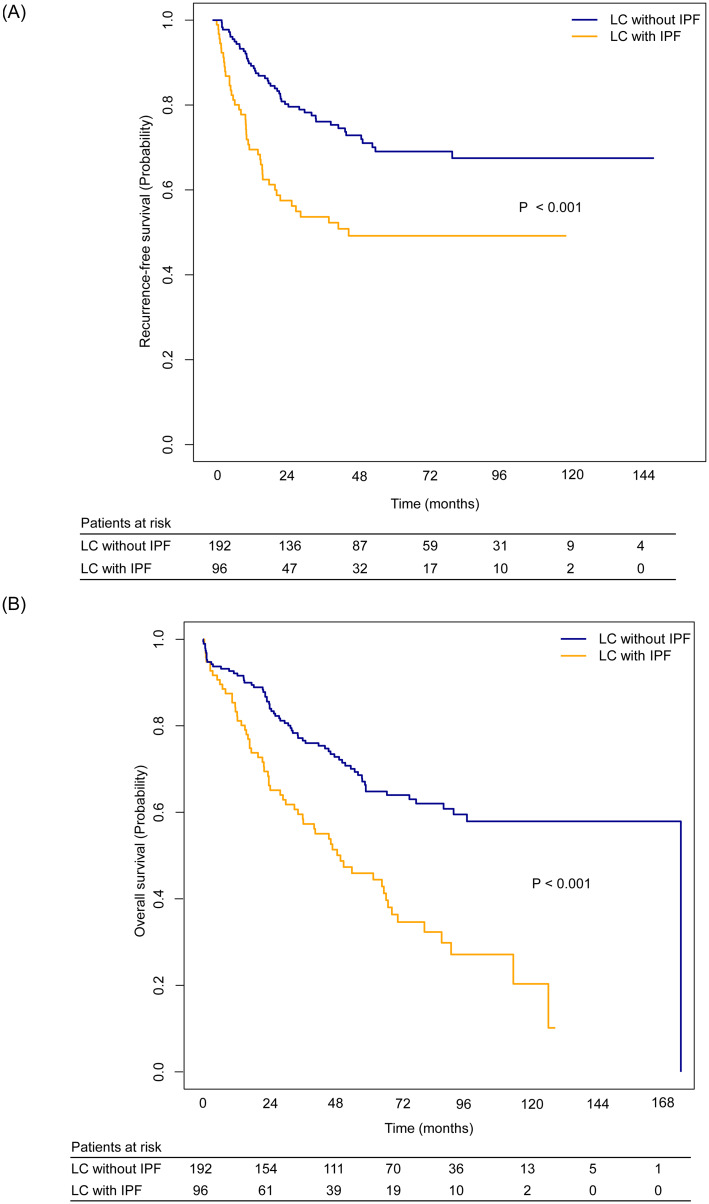
Probability of recurrence-free survival and overall survival for patients with surgically resected non-small cell lung cancer with or without IPF. (A) Recurrence-free survival was significantly lower in the LC with IPF group than in the LC without IPF (P<0.001). (B) Overall survival was significantly lower in the LC with IPF group than in the LC without IPF (P<0.001).

During a median follow-up period of 49.2 months, 92 recurrences were documented for the entire matched study population. Recurrences were significantly more frequent in the LC with IPF group than in the LC without IPF group (44.8% vs. 25.5%; P = 0.002). Among the patients with recurrence, the type of recurrence (local, regional recurrence or distant metastasis) did not show significant difference between groups. The 5-year recurrence-free survival rate (49.2%; 95% CI, 39.4–61.4 vs. 69.1%; 95% CI, 61.9–77.0; P<0.001; Fig 2, Table 5) and 5-year overall survival rate (45.9%; 95% CI, 36.5–57.7 vs. 64.8%; 95% CI, 57.9–72.6; P<0.001; Fig 2, Table 5) were significantly lower in the LC with IPF group than in the LC without IPF group. Cancer-specific survival was also significantly lower in the LC with IPF group than in the LC without IPF group (P = 0.008, S1A Fig, Table 5).

**Table 5:** Recurrence and overall survival in patients with surgically resected non-small cell lung cancer with or without idiopathic pulmonary fibrosis.

	LC with IPF (n = 96)	LC without IPF (n = 192)	P-value
Recurrence	43 (44.8)	49 (25.5)	0.002
Type of recurrence			0.062
Local recurrence	11 (25.6%)	4 (8.2%)	
Regional recurrence	7 (16.3%)	13 (26.5%)	
Distant metastasis	25 (58.1%)	32 (65.3)	
5-year recurrence-free survival rate (%), 95% CI	49.2 (39.4–61.4)	69.1 (61.9–77.0)	<0.001
Recurrence-free survival (months), 95% CI	48.8 (23.0–NA)	NA	
Death	61 (63.5%)	67 (34.9%)	<0.001
Cause of death			
Lung cancer progression	30 (31.2%)	36 (18.8%)	
Surgery-related complication*	5 (5.2%)	9 (4.7%)	
IPF progression or acute exacerbation	10 (10.4%)	NA	
Pneumonia	9 (9.4%)	8 (4.2%)	
Other**	7 (2.4%)	14 (7.3%)	
5-year survival rate (%), 95% CI	45.9 (36.5–57.7)	64.8 (57.9–72.6)	<0.001
Overall survival (months), 95% CI	49.5 (35.9–68.0)	172.0 (NA–NA)	
Cancer specific survival (months), 95% CI	89.3 (68.0–NA)	172.0 (NA–NA)	0.008

Values are expressed as number (%).

IPF, idiopathic pulmonary fibrosis; LC, lung cancer

* The duration between surgery and the onset of complications was ≤4 weeks

** 1 heart failure, 1 stomach perforation, 3 sepsis, 3 other cancer, 13 unknown

### Predictors of recurrence

Univariable Cox proportional hazards regression analysis revealed that IPF, the postoperative stage, surgical extent, and rate of mediastinal lymph node dissection were major predictors of recurrence after surgical treatment. These variables and additional potential confounding variable were combined in multivariable regression analysis, which revealed that IPF (HR, 2.68; 95% CI, 1.53–5.76; P = 0.001) and postoperative stage III (vs. stage I; HR, 5.46; 95% CI, 1.72–17.37; P = 0.004) were independent predictors of recurrence (Table 6).

**Table 6:** Stratified Cox regression analyses for recurrence and mortality after surgical treatment of non-small cell lung cancer in patients with or without idiopathic pulmonary fibrosis (propensity score-matched population).

(A) Stratified Cox regression analyses for recurrence						
	Univariable			Multivariable		
	HR	95%CI	p-value	HR	95%CI	p-value
IPF	2.86	1.72–4.76	<0.001	2.68	1.53–5.76	0.001
Sex	2.34	0.83–6.59	0.108			
Age	0.97	0.92–1.02	0.236			
Smoking (pack-years)	0.99	0.98–1.00	0.173			
ECOG ≥2	5.16	0.52–50.41	0.158			
FVC % pred	1.00	0.98–1.01	0.640			
FEV_1_% pred	1.00	0.99–1.02	0.527			
Postoperative Stage						
I	1.00					
II	2.00	0.91–4.39	0.084	2.39	0.78–7.30	0.339
III	5.19	1.98–13.64	<0.001	5.46	1.72–17.37	0.004
Surgery extent						
Wedge resection	1.00					
Segmentectomy	0.30	0.06–1.59	0.158	0.85	0.11–6.85	0.882
Lobectomy	0.26	0.07–0.93	0.038	1.36	0.23–8.07	0.738
Biolobectomy	0.22	0.04–1.17	0.076	1.64	0.17–16.05	0.670
Pneumonectomy	0.77	0.12–4.83	0.780	3.56	0.30–41.80	0.313
MLND	0.17	0.05–0.65	0.009	0.24	0.04–1.70	0.154
Dissected LN (n)	0.99	0.96–1.01	0.257			
Resection margin	1.47	0.44–4.86	0.529			
Adjuvant chemotherapy*	1.28	0.72–2.27	0.400	0.95	0.42–2.18	0.905
(B) Stratified Cox regression analyses for mortality						
	Univariable			Multivariable		
	HR	95%CI	p-value	HR	95%CI	p-value
IPF	2.38	1.56–3.64	<0.001	1.98	1.12–3.47	0.018
Sex	0.81	0.33–2.00	0.652			
Age	0.97	0.93–1.02	0.227			
Smoking (pack-years)	1.00	1.00–1.01	0.722			
ECOG ≥2	5.16	1.03–25.86	0.046	1.73	0.90–3.33	0.101
FVC % pred	0.98	0.97–1.00	0.049	0.98	0.96–1.01	0.219
FEV_1_% pred	0.99	0.98–1.00	0.113			
Postoperative Stage						
I	1.00					
II	1.49	0.81–2.73	0.196	1.83	0.79–4.23	0.158
III	3.56	1.67–7.60	<0.001	3.39	1.28–8.97	0.014
Surgery extent						
Wedge resection	1.00					
Segmentectomy	1.02	0.22–4.64	0.982	1.01	0.17–5.92	0.993
Lobectomy	0.27	0.09–0.80	0.018	0.41	0.10–1.61	0.200
Biolobectomy	0.55	0.16–1.90	0.345	0.86	0.18–4.14	0.848
Pneumonectomy	1.67	0.38–7.25	0.496	2.43	0.39–15.31	0.344
MLND	0.60	0.21–1.68	0.330			
Dissected LN (n)	0.99	0.97–1.01	0.259			
Resection margin	1.00	0.36–2.78	1.000			
AE+ARDS	16.00	2.00–127.90	0.009	14.02	1.55–126.89	0.019
Adjuvant chemotherapy*	0.94	0.56–1.58	0.830	0.92	0.45–1.88	0.810

AE or ARDS, acute exacerbation of idiopathic pulmonary fibrosis or acute respiratory distress syndrome; DL_CO_, diffusing capacity of the lungs for carbon monoxide; ECOG, Eastern Cooperative Oncology Group; FVC, forced vital capacity; FEV_1_, Forced expiratory volume in one second; MLND, modified lymph node resection; LN, lymph node; IPF, idiopathic pulmonary fibrosis

*Use of any chemotherapy including chemotherapy, Concurrent chemoradiotherapy, Sequential radiotherapy after chemotherapy

### Predictors of overall survival

Univariable Cox proportional hazards regression analysis revealed that IPF, Eastern Cooperative Oncology Group score, FVC, postoperative stage, surgical extent, and surgery-related AE-IPF or ARDS were major covariates for overall survival. These variables and additional potential confounding variable were combined in multivariable analysis, which revealed that IPF (HR, 1.98; 95% CI, 1.12–3.47; P = 0.018), postoperative stage III NSCLC (vs. stage I; HR, 3.39; 95% CI, 1.28–8.97; P = 0.014), and surgery-related AE-IPF or ARDS (HR, 14.02; 95% CI, 1.55–126.89; P = 0.019) were independent predictors of overall survival (Table 6).

## Discussion

In the present study, we demonstrated the impact of IPF on recurrence and survival following curative surgical resection of stage I–III NSCLC in a propensity score-matched study population. Both recurrence-free survival and overall survival after curative surgical resection were significantly compromised in patients with IPF. IPF and postoperative stage III were predictors of both recurrence and overall survival. Surgery-related AE-IPF or ARDS was also identified as an independent risk factor for mortality.

Epidemiological studies have revealed that IPF is an independent risk factor for LC development [4, 6, 11, 23], although the pathogenesis connecting the two diseases remains poorly understood. Common pathways for the development of LC in IPF, yet with distinct roles in the pathogenesis of both conditions, include genetic and epigenetic alterations; abnormal expression of microRNAs; cellular and molecular aberrances; and activation of specific signaling transduction pathways [24–26]. Several pieces of evidence related to therapeutic agents also suggest a pathogenic link between IPF and LC. Nintedanib, which was first approved for use in combination with docetaxel for the second-line treatment of advanced NSCLC [27], has also been approved as an antifibrotic agent for the treatment of IPF. A previous retrospective study reported that the incidence of LC was lower in patients with IPF receiving pirfenidone, another antifibrotic agent approved for IPF treatment, than in those without pirfenidone treatment [28], while a preclinical experiment showed that the combination of pirfenidone and cisplatin led to increased apoptosis and synergistic death of both cancer-associated fibroblasts and NSCLC cells [29].

Patients with LC and underlying IPF are commonly encountered in clinical practice; however, for these patients, it is difficult to predict the efficacy of treatment for LC due to the increased incidence of treatment-related complications and poor prognosis of IPF itself [12–14, 30]. Reports on the long-term prognosis of patients with NSCLC accompanied by IPF who underwent curative surgical resection have consistently documented lower 5-year survival and recurrence-free survival for patients with IPF than for those without [14, 17–20]. In a study performed by the Japanese Association for Chest Surgery, which analyzed predictors of overall survival after the surgical treatment of LC in patients with interstitial lung disease [31], it was found that wedge resection, a predicted FVC of <80%, and tumors located in the inferior lobe were poor prognostic factors for overall survival. Furthermore, subgroup analysis of patients with stage I cancer showed that the survival curves for those treated with wedge resection and those treated with lobectomy crossed at approximately 1 year after surgery, with 5-year survival rates of 29.2% and 68.6%, respectively. From this result, the authors inferred that wedge resection is associated with a lower incidence of surgery-related complications and better short-term outcomes than is lobectomy, although the long-term outcomes are worse with wedge resection, probably due to a higher recurrence rate. In the present study, however, multivariable analysis for recurrence-free survival showed that IPF and postoperative stage III were independent risk factors for recurrence after adjustment for other variables, including the surgical extent. In order to confirm that inferior recurrence-free survival and overall survival in the LC with IPF group were not due to the difference in surgical extent between groups, subgroup analysis was performed for patients who underwent lobectomy in the propensity score matched population. Subgroup analysis showed that recurrence-free survival, overall survival and cancer-specific survival were significantly inferior in the LC with IPF group than in the LC without IPF group (S1 and S2 Figs).

Unlike previous studies on patients with NSCLC and IPF, we assessed detailed surgery-related variables, including whether or not mediastinal lymph node dissection was performed, the number of dissected LNs and resection margin status. However, none were found to be significant predictors of recurrence-free survival. Therefore, IPF itself appears to increase the risk of lung carcinogenesis and recurrence after curative resection. This may be explained by the aforementioned pathways for carcinogenesis in IPF, along with the possibility that tumors that developed within fibrotic lesions could be obscured by honeycombing or reticular changes in fibrotic lesions and consequently overlooked. Moreover, the probability of undiscovered positive LNs is higher for patients with IPF; this can be explained by the lower number of dissected LNs in our IPF group, which could lead to stage migration and remnant malignancy [32].

Previous studies showed that morbidity and mortality rates after the surgical treatment of NSCLC were significantly higher in patients with IPF than in those without IPF, and inferred that AE-IPF is the main cause of morbidity and mortality [12–14, 30]. The present study also found a higher incidence of surgery-related AE-IPF or ARDS in the LC with IPF group. Multivariable analysis for overall survival reconfirmed that surgery-related AE-IPF or ARDS, along with IPF itself and postoperative stage III, was an independent risk factor for overall survival. Therefore, close monitoring of postoperative respiratory complications in patients with IPF is important. DL_CO_, which showed a significant difference between the LC with IPF and LC without IPF groups in the propensity score-matched population, was excluded from the Cox regression model due to 39 patients with missing DL_CO_ data in the LC without IPF group. The DL_CO_ test was mainly missed in patients without underlying lung fibrosis and respiratory symptoms before surgery. Nevertheless, since DL_CO_ has been reported as a risk factor for postoperative respiratory complication, patients with a low DL_CO_ should be carefully evaluated, particularly for surgery-related AE-IPF or ARDS [33].

This study had several limitations. First, as the surgical extent was not included in propensity score matching due to lack of data, the LC with IPF group received sublobar resection more frequently compared to the LC without IPF group in the propensity matched population, which can be a risk factor for recurrence. To cope with these limitations, stratified Cox regression analysis was conducted including the surgical extent, and it revealed that IPF and cancer stage were independent risk factor for recurrence regardless of surgical extent. Second, more detailed surgical and tumor variables that can affect recurrence, including the distance from the tumor to the resection margin, visceral pleural invasion, angiolymphatic invasion, and the degree of tumor differentiation, were not available for analysis. Lastly, this study had a retrospective nonrandomized design, which is associated with various biases and confounding factors. However, we obtained the data from two tertiary care hospitals to minimize biases. Further studies with a larger patient sample are required to validate our findings.

## Conclusions

The present study revealed that the recurrence-free survival rate after curative surgical resection of stage I–III NSCLC was significantly lower for patients with IPF than for those without IPF, and that IPF and postoperative stage III were predictors of both recurrence and mortality. These findings suggest that close surveillance for recurrence after curative surgical resection of NSCLC is mandatory if the patient has underlying IPF. We suggest that the conventional regimen involving follow-up computed tomography every 6 months after surgery should be replaced with a regimen involving follow-up visits at shorter intervals in patients with underlying IPF, regardless of the surgical extent especially in higher cancer stage.

## Supporting information

S1 Fig Probability of cancer specific survival for patients with surgically resected non-small cell lung cancer with or without IPF. (A) Cancer-specific survival was significantly inferior in the LC with IPF group compared to the LC without IPF group in the whole propensity score matched population (P = 0.008). (B) Cancer-specific survival was significantly inferior in the LC with IPF group compared to the LC without IPF group in patients who underwent lobectomy among the propensity score-matched population (P = 0.007).
(PPTX)

S2 Fig Probability of recurrence-free survival and overall survival in patients who underwent lobectomy among propensity score-matched population. (A) Recurrence-free survival was significantly inferior in the LC with IPF group than in the LC without IPF group (P = 0.003). (B) Overall survival was also significantly inferior in the LC with IPF group than in the LC without IPF group (P<0.001).
(PPTX)

S1 Data Relevant data. (XLSX)

## Abbreviations

AE-IPF: acute exacerbation of IPF
ARDS: acute respiratory distress syndrome
CI: confidence interval
DL_CO_: diffusing capacity of the lungs for carbon monoxide
FVC: forced vital capacity
HR: hazard ratio
IPF: idiopathic pulmonary fibrosis
LC: lung cancer
NSCLC: non-small cell lung cancer
