# Supplementary material for: Impact of idiopathic pulmonary fibrosis on recurrence after surgical treatment for stage I–III non-small cell lung cancer
Source: PLoS One. 2020 Jun 29;15(6):e0235126. doi: 10.1371/journal.pone.0235126 (PMC7323957; doi:10.1371/journal.pone.0235126)
Supplement: S1 Fig — (A) Cancer-specific survival was significantly inferior in the LC with IPF group compared to the LC without IPF group in the whole propensity score matched population (P = 0.008). (B) Cancer-specific survival was significantly inferior in the LC with IPF group compared to the LC without IPF group in patients who underwent lobectomy among the propensity score-matched population (P = 0.007). (PPTX) [file pone.0235126.s001.pptx]

## Slide 1
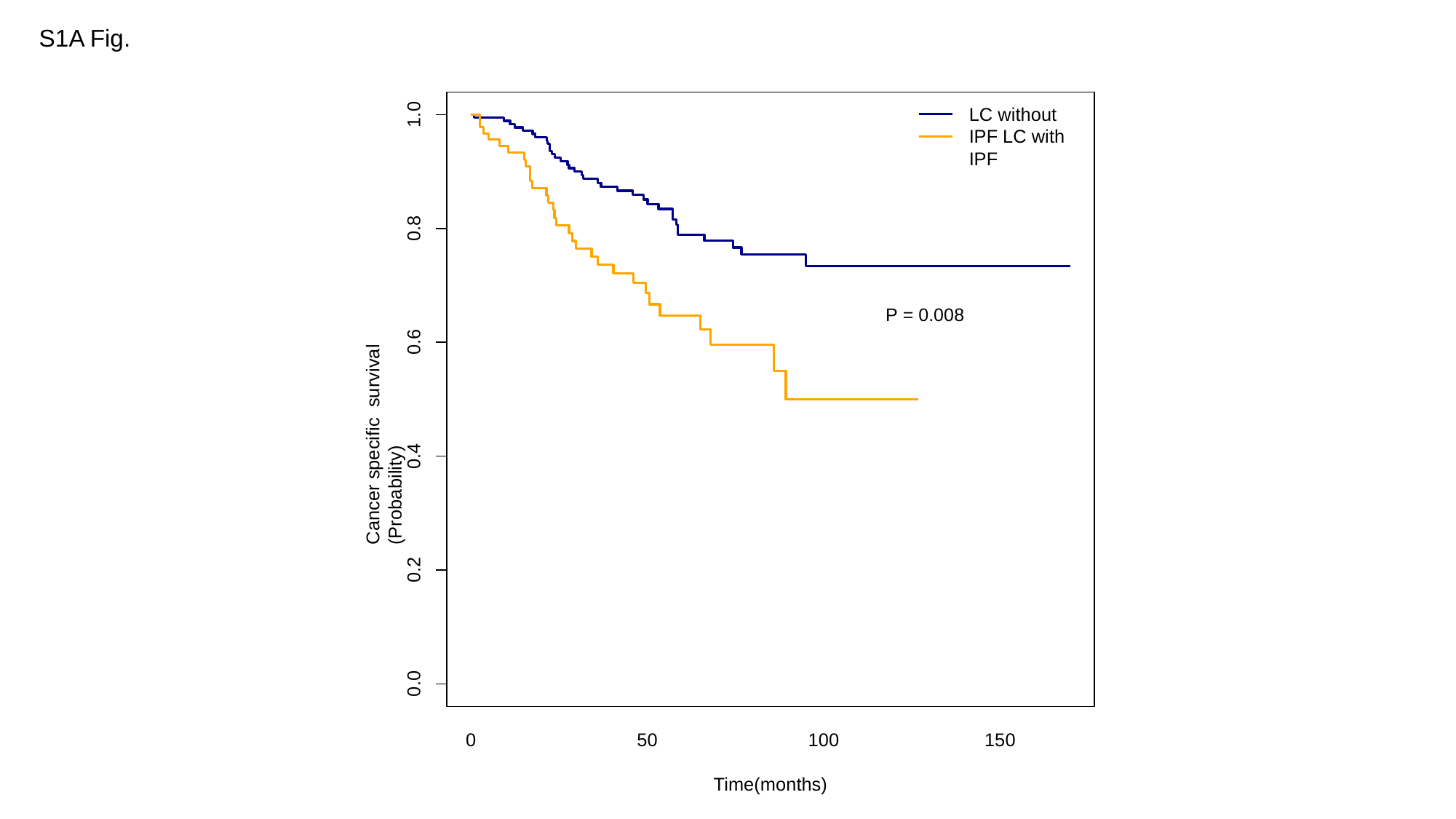

S1A Fig.
1.0
LC without IPF LC with IPF
0.8
Cancer specific survival (Probability)
P = 0.008
0.6
0.4
0.2
0.0
0
50
100
150
Time(months)

## Slide 2
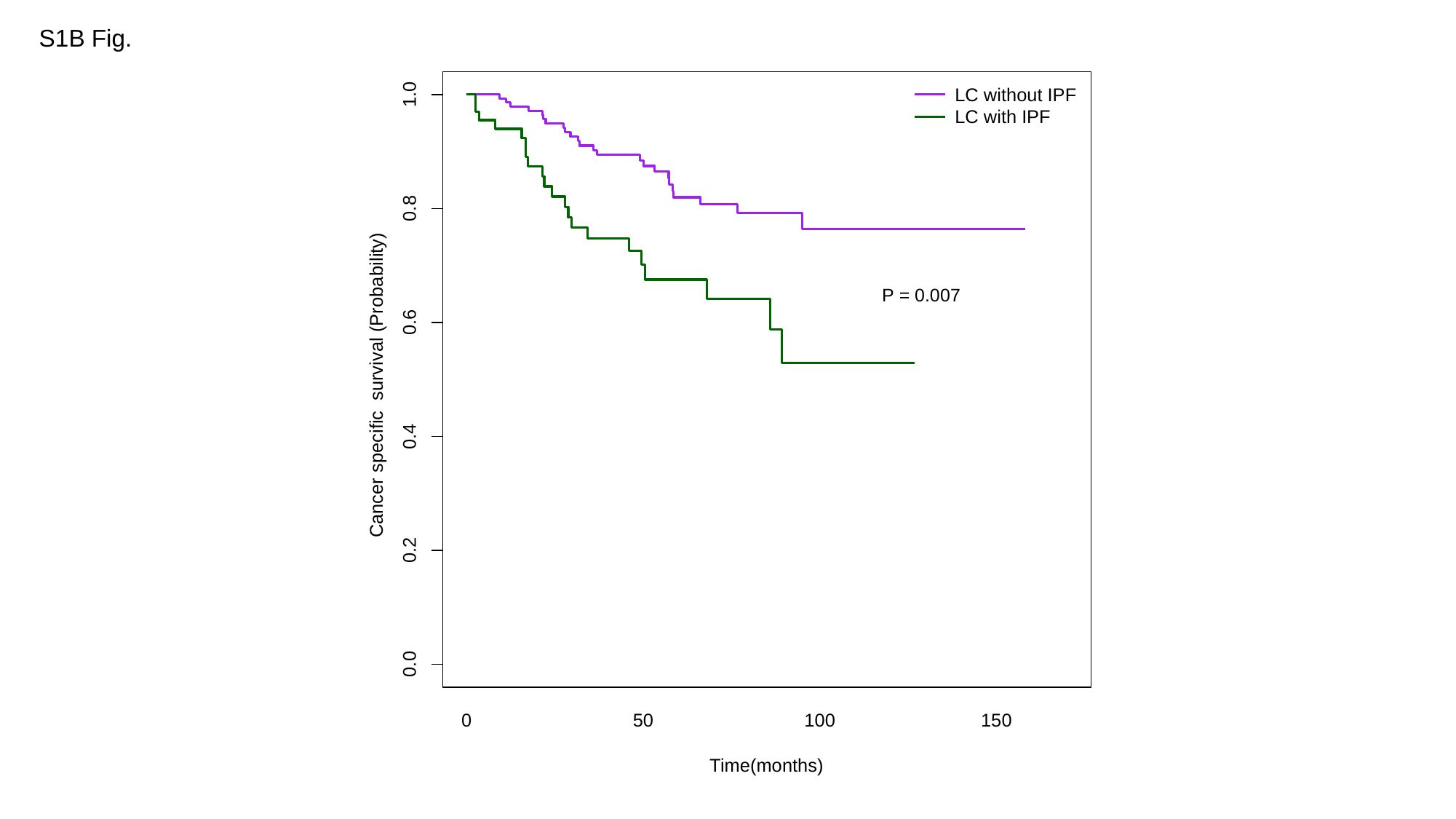

S1B Fig.
1.0
LC without IPF LC with IPF
0.8
Cancer specific survival (Probability)
P = 0.007
0.6
0.4
0.2
0.0
0
50
100
150
Time(months)
