# Supplementary material for: Impact of idiopathic pulmonary fibrosis on recurrence after surgical treatment for stage I–III non-small cell lung cancer
Source: PLoS One. 2020 Jun 29;15(6):e0235126. doi: 10.1371/journal.pone.0235126 (PMC7323957; doi:10.1371/journal.pone.0235126)
Supplement: S2 Fig — (A) Recurrence-free survival was significantly inferior in the LC with IPF group than in the LC without IPF group (P = 0.003). (B) Overall survival was also significantly inferior in the LC with IPF group than in the LC without IPF group (P<0.001). (PPTX) [file pone.0235126.s002.pptx]

## Slide 1
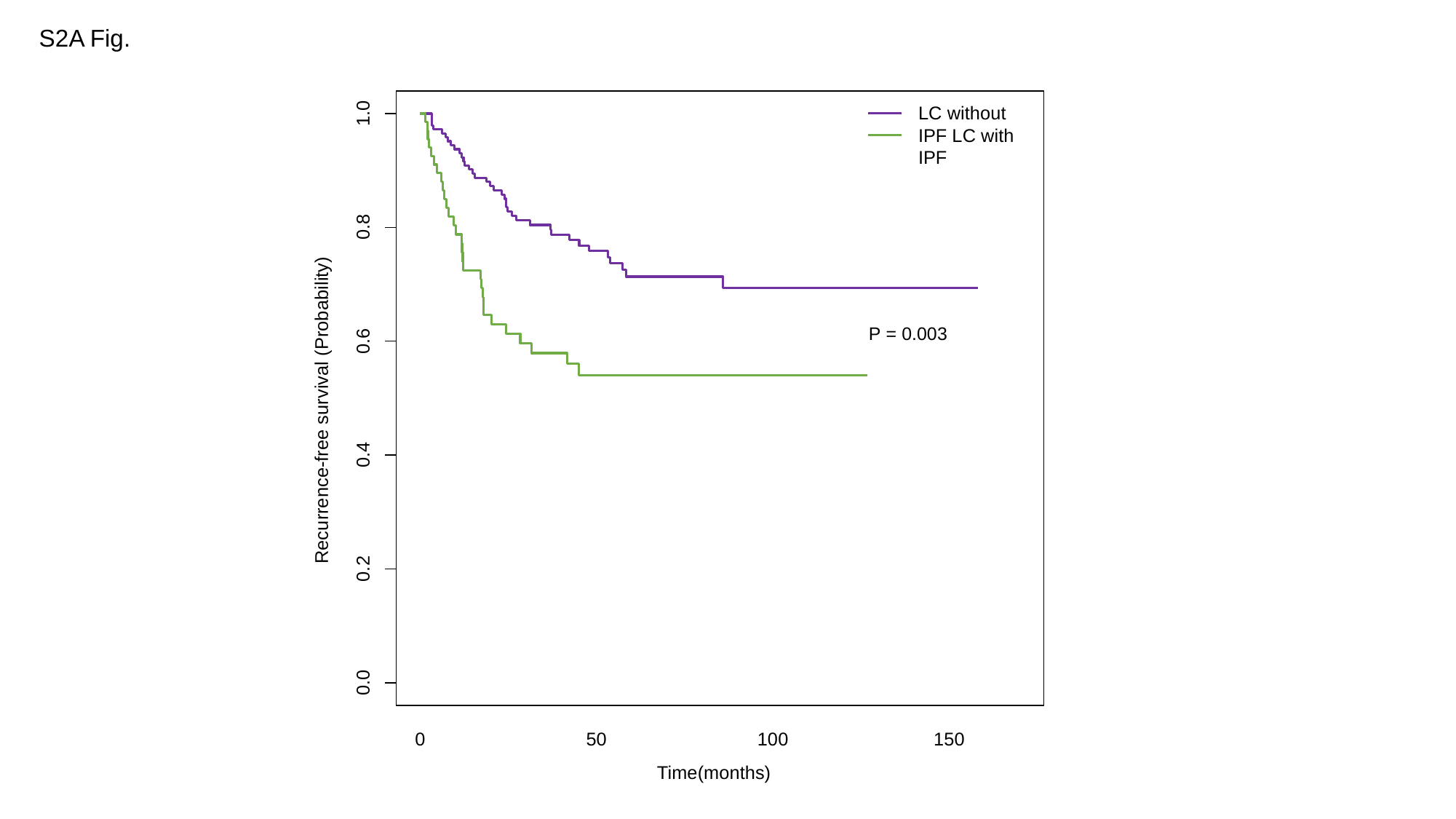

S2A Fig.
LC without IPF LC with IPF
1.0
0.8
0.6
0.4
0.0
0.2
Recurrence-free survival (Probability)
0
50
100
150
Time(months)
P = 0.003

## Slide 2
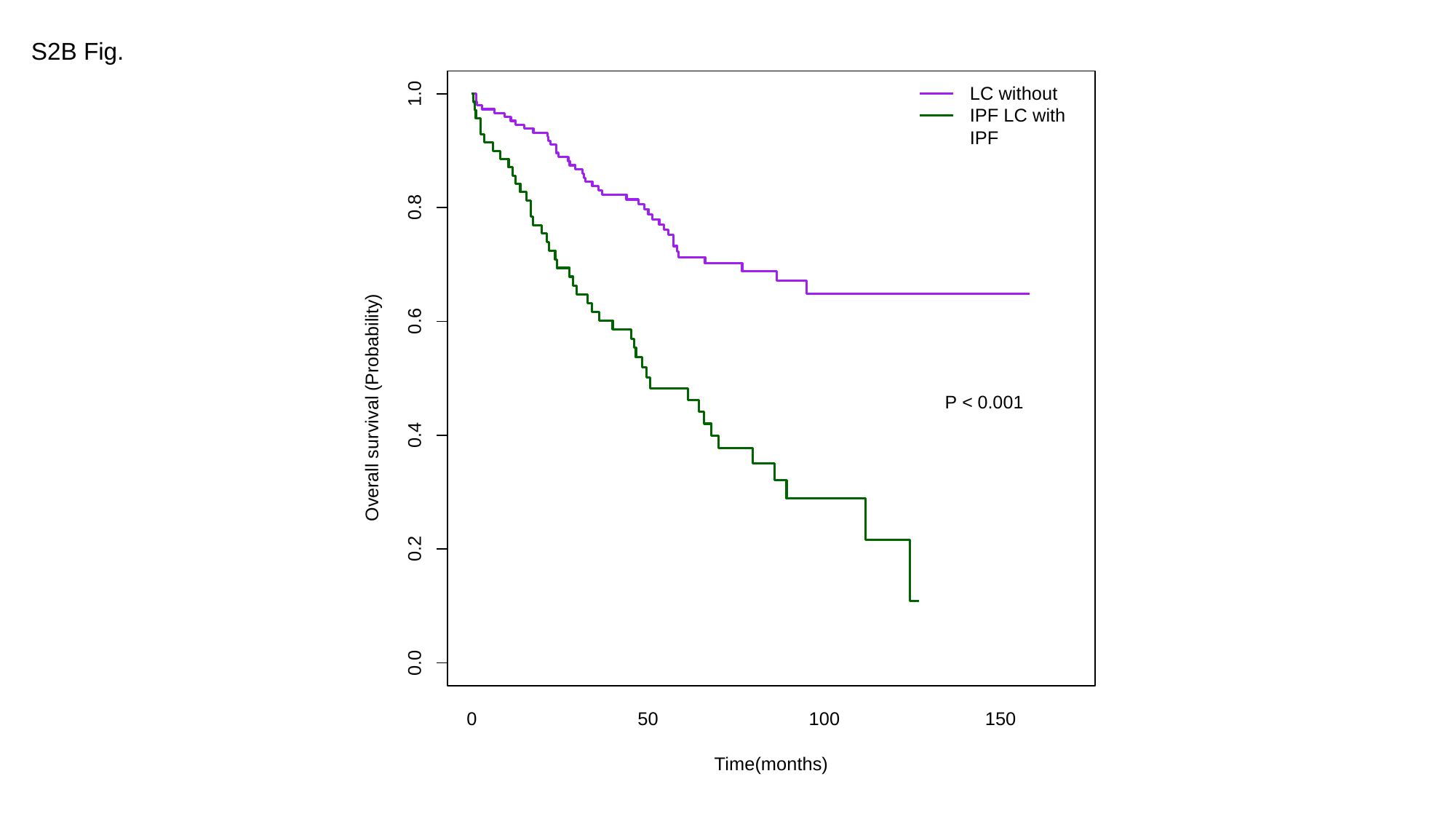

S2B Fig.
LC without IPF LC with IPF
0.8
Overall survival (Probability)
P < 0.001
0.4
0
50
100
150
Time(months)
1.0
0.6
0.2
0.0
